# Electromyographic Analysis of the Lumbar Extensor Muscles during Dynamic Exercise on a Home Exercise Device

**DOI:** 10.3390/jfmk7010026

**Published:** 2022-03-01

**Authors:** John M. Mayer, Brian E. Udermann, Joe L. Verna

**Affiliations:** 1 The Vert Mooney Research Foundation (DBA US Spine & Sport Foundation), San Diego, CA 92111, USA; joev@spineandsport.com; 2 Department of Exercise and Sport Science, College of Science and Health, University of Wisconsin—LaCrosse, LaCrosse, WI 54601, USA; budermann@uwlax.edu; 3 Spine & Sport Physical Therapy Inc., San Diego, CA 92111, USA

**Keywords:** low back pain, electromyography, exercise training, home exercise program, virtual care

## Abstract

Resistance exercise with devices offering mechanisms to isolate the lumbar spine is effective to improve muscle strength and clinical outcomes. However, previously assessed devices with these mechanisms are not conducive for home exercise programs. The purpose of this study was to assess the surface electromyographic (EMG) activity of the lumbar extensor muscles during dynamic exercise on a home back extension exercise device. Ten adults (5 F, 5 M) performed dynamic lumbar extension exercise on a home device at three loads: 1.00 × body weight (BW), 1.25 × BW and 1.50 × BW. Surface EMG activity from the L3/4 paraspinal region was collected. The effect of exercise load, phase of movement, and position in the range of motion on lumbar extensor EMG activity (normalized to % maximum voluntary isometric contraction) was assessed. Lumbar extensor EMG activity significantly increased from 1.00 BW to 1.50 BW loads (*p* = 0.0006), eccentric to concentric phases (*p* < 0.0001), and flexion to extension positions (*p* < 0.0001). Exercise using a home back extension exercise device progressively activates the lumbar extensor muscles. This device can be used for home-based resistance exercise programs in community-dwelling adults without contraindications.

## 1. Introduction

Low back pain is common, costly, disabling, and greatly impacts the quality of life of adults around the world [1,2]. Hundreds of treatment options are available for low back pain, many of which have only modest results [2]. Physical exercise is typically recommended as an effective prevention and treatment strategy for low back pain [2]. Among the options for therapeutic exercise, progressive resistance exercise using back extension machines that isolate the lumbar spine has a relatively large body of evidence to support safety, and ability to improve physical function and reduce disability [3].

The premise behind use of these machines is that isolating the lumbar spine through various restraint mechanisms forces the lumbar extensor muscles (e.g., multifidus, erector spinae) to be the primary producers of torque during compound trunk extension, thereby limiting the input of the more powerful gluteal and hamstring muscles [4,5]. This strategy has been shown to effectively activate the lumbar extensors and other trunk extensor muscles [6,7]. Progressive resistance exercise training on these devices has been shown to result in large strength gains in healthy adults [4,5], and relieve symptoms and restore functional capacity in individuals with chronic low back pain [3,8,9,10]. Existing back extension machines that stabilize the pelvis in the seated position and allow for gradual loading of lumbar extensors are intended for in-clinic use, which requires face-to-face interactions with a therapist or trainer. The computerized lumbar extension dynamometer (MedX, Altamonte Springs, FL, USA) is an example of such a machine. Despite its benefits, it is relatively costly, is not portable, has a large footprint [3], and is not intended for home use. Therefore, the development of portable, cost-effective, and efficacious back extension exercise devices is needed to foster home exercise programs.

The demand for implementation of virtual solutions to deliver exercise programs for the management of low back pain is increasing, and the COVID-19 pandemic has provided an impetus to explore such solutions. A recent systematic review found that telehealth is safe and effective for the management of non-acute low back pain [11]. A recent observational study found that virtual care focusing on exercise for managing low back pain resulted in similar improvements in function and pain reduction compared to in-clinic programs [12]. Moreover, another recent observational study found benefits of virtual physical therapy in terms of patient satisfaction and improving access to care [13]. However, the overall body of evidence on virtual care is minimal and no standard exists for home-based exercise delivery, particularly for programs focusing on progressive resistance exercise training. Thus, implementation of progressive resistance exercise programs delivered virtually has been limited for managing low back pain.

**Figure 1:**
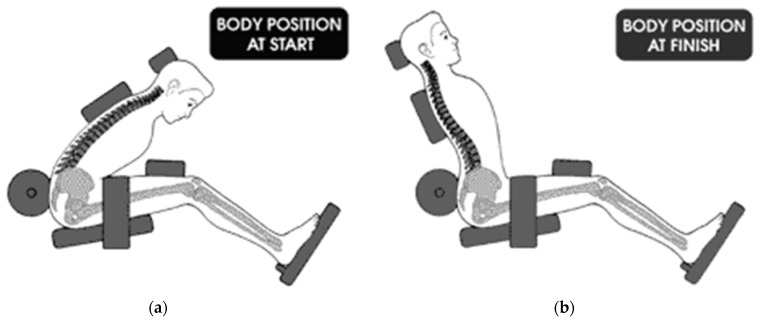
Illustration of pelvic restraint mechanisms and movement patterns on the home back extension exercise device. (**a**) Start position (lumbar flexion). (**b**) Finish position (lumbar extension).

**Figure 2:**
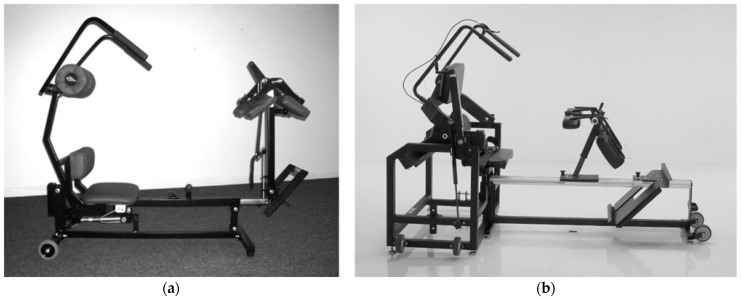
Home Back Extension Exercise Device (MedX Home Back Device, Converge Medical Technology LLC., Austin, TX, USA). (**a**) Prototype assessed in study. (**b**) Current version.

A smaller, portable, and less costly back extension exercise device (MedX Home Back Device, Converge Medical Technology LLC, Austin, TX, USA) was developed as an alternative to provide isolated progressive resistance exercise for the lumbar extensors. The device is intended for home use and is not difficult to implement, administer, and complete exercise sessions. It utilizes body weight and external loads (metal plates—assessed prototype; resistance bands or hydraulics: current version) to apply resistive loading during back extension exercise in the seated position. It also incorporates similar mechanisms to isolate the lumbar spine as the computerized lumbar extension dynamometer (Figure 1 and Figure 2). However, the ability of the home back extension exercise device to effectively activate the lumbar extensor muscles and provide progressive loads has not been explored. Therefore, the purpose of this study was to assess the surface electromyographic (EMG) activity of the lumbar extensor muscles during full range of motion, dynamic exercise on a home back extension exercise device at three exercise loads.

## 2. Materials and Methods

### 2.1. Study Design

An observational study with repeated measures was conducted at a clinical facility. Participants reported to the facility on one occasion during which physical performance measures and surface EMG data were collected multiple times.

### 2.2. Participants

A convenience sample of participants were recruited by word of mouth and posted flyers to include an equal number of males and females. The study’s protocol was reviewed and approved by Biomed IRB (San Diego, CA, USA) and each participant provided written informed consent. Inclusion criteria for participation were [7]: 18–45 years of age; good general health; ability to provide written informed consent. Exclusion criteria were: History of significant clinical low back pain; history of lumbar spine pathology, deformity, or surgery; knee or hip disorders contraindicating use of the exercise testing device’s pelvic restraint mechanisms; cardiovascular or other orthopedic contraindications to resistance exercise; a “yes” response for any item on the physical activity readiness questionnaire at screening [14]; history of high blood pressure; resting blood pressure and heart rate measurements outside of the normal range at screening; current participation in a resistance exercise program for the back musculature; pregnant females.

### 2.3. Sample Size Calculation

Sample size was estimated using G*Power 3.1 [15], based on previous work assessing lumbar extensor muscle surface EMG activity during exercise [7,16,17] and the following parameters: 25% increase in mean value from exercise at 1.00 × body weight (BW) to 1.50 × BW with a standard deviation of approximately 50% of the mean value (effect size = 0.50), repeated measures, power = 0.80, alpha = 0.05. Based on these parameters, a sample size of *n* = 10 was adequate.

### 2.4. Procedures

#### 2.4.1. Participant Selection and Screening

Candidates who responded to recruitment efforts contacted the investigator by telephone. To confirm eligibility, a standardized telephone screening questionnaire was used. Candidates who were eligible according to the telephone screen were referred to the study site to complete additional screening procedures. After providing informed consent, candidates completed a health history questionnaire and the physical activity readiness questionnaire [14]. Next, resting blood pressure and heart rate were recorded from eligible candidates. Finally, females were administered a urine pregnancy test. At this time, eligible candidates were invited to participate in the study.

#### 2.4.2. Assessment of Isometric Lumbar Extension Strength

Immediately following the screening procedures, height and body weight were recorded from eligible participants. Next, isometric lumbar extension strength was assessed, which was used to normalize EMG data as a percentage of Maximum Voluntary Isometric Contraction (%MVIC). For the strength test, the participant was seated in an upright position on a computerized lumbar extension dynamometer (MedX Corp.) with its pelvic restraint mechanisms engaged (e.g., lap belt, femur restraint pad, pelvic restraint pad, footboard). In the seated testing position, the participant was upright with the hips flexed at approximately 70–80 degrees and in slight internal rotation, and the knees flexed at approximately 20 degrees. The participant performed two trials of strength tests on the device—submaximal test and maximal test. After establishing the test position, the participant performed light dynamic exercise and submaximal isometric strength tests in the sagittal plane for familiarization to the isometric testing and dynamic exercise procedures. For the submaximal isometric strength tests, the participant pushed against the thoracic pad on the device while using moderate effort. Submaximal testing was performed one time at three positions—72, 36, and 0 degrees of lumbar flexion, which represents the full range of motion in the sagittal plane allowed by the testing device. After a 15-min rest, the participant performed the actual tests to determine maximum voluntary isometric lumbar extension torque at the same three positions that were used for the submaximal testing. At each position, the participant gradually built up force against the thoracic pad (using the trunk extensor muscles) and pushed as hard as possible for approximately one second. A monitor provided visual feedback of performance and the investigator provided verbal encouragement for the participant to generate maximum force. Isometric strength (torque) was recorded electronically by the device in foot-pounds (ft-lb) and converted to Newton-meters (N-m). This isometric strength testing protocol has been validated and described in detail [7,18].

#### 2.4.3. Assessment of Dynamic Lumbar Extension Exercise

Following the strength test and a 15-min rest, the participants completed 1 set of full range of motion dynamic exercise on the prototype version of the home back extension exercise device (Figure 2A) at 3 exercise loads (3 sets total—1 set at each of 3 exercise loads), with a 3-min rest between each set. The order of exercise at the 3 loads was balanced across participants. The exercise loads for the 3 sets were 1.00 times body weight (BW), 1.25 BW, and 1.50 BW. Loads greater than body weight were accommodated by metal plates that were attached to the device.

Each set consisted of three repetitions using a slow movement exercise protocol (i.e., 10 s concentric, 10 s eccentric). For each set of dynamic exercise, the participant was positioned at full flexion on the device and completed the concentric phase by extending their low back against the thoracic pad until reaching full pain-free extension. Upon reaching full extension, the participant completed the eccentric phase by slowly returning to the starting position. Three repetitions were completed for each set. To standardize the tempo of the movement, a metronome was used and was set at 60 beats per minute. Information about adverse events (e.g., muscle soreness) was gathered through verbal subjective reports from the participants during and after lumbar extension strength tests and dynamic exercises on the study visit, and during four subsequent days until symptoms resolved.

#### 2.4.4. Instrumentation and EMG Processing

Surface EMG signals were collected from the right and left lumbar paraspinal region at the L3–4 level during the isometric strength test and each set of dynamic exercise utilizing techniques adapted from previous work [7,16]. First, the skin was palpated to establish landmarks for the regions of interest and was scrubbed with an alcohol pad. Two round (1.5 cm) self-adhesive, disposable silver/silver chloride, pre-gelled, snap surface electrodes were placed on the skin surface. The location of electrode placement was 1 cm above and below the L3–4 interspace over the central portion of the paraspinal muscle belly bilaterally, which was approximately 2–3 cm from the midline of the spine in the sagittal plane. Active electrodes were used according to the manufacturer’s recommendations (Noraxon USA Inc., Scottsdale, AZ, USA) [19,20], and the inter-electrode distance was 2 cm.

Electromyographic signals were collected with a 1000 Hz sampling rate. Characteristics of the differential amplifier were as follows according to the manufacturer’s recommendations [19,20]: bandpass filter: high pass cutoff—10 Hz, low pass cutoff—500 Hz; common mode rejection: minimum 85 dB at 1000 Hz; input impedance: >10 Megaohm. Prior to processing, raw EMG data were visually inspected in order to detect noise (e.g., mechanical or movement artifacts, electrical signals from other sources, such as electrocardiograms and power lines). Raw EMG data were rectified, smoothed (via root mean square (RMS) technique with 50 ms interval), filtered (median 5 filtering technique), and normalized (based on MVIC values obtained from the strength test). Myoresearch v.2.1 software (Noraxon USA Inc.) was used for EMG data processing and analysis. The reliability of EMG data collected in a similar manner was shown to be acceptable [7].

### 2.5. Outcome Measures

The primary outcome measure was lumbar extensor muscle surface EMG activity expressed as the normalized value in %MVIC. Data obtained from the second repetition of exercise were used for analysis. The second repetition of exercise was arbitrarily selected to minimize potential artifacts in muscle activity due to acceleration in the concentric phase to start the exercise set (i.e., first repetition at its start point) and deceleration at the eccentric phase to end the exercise set (i.e., third repetition at its end point). Thus, the second repetition was the most likely repetition to be performed in the desired smooth, controlled fashion without artifacts. Data were normalized using a previously published method [7], using the following equation:

%MVIC = (Raw EMG (mV/sec) dynamic exercise/Raw EMG (mV/sec) peak isometric contraction) × 100%

### 2.6. Data Analysis

Descriptive data (group means and standard deviations) were calculated for normalized EMG (in %MVIC) by exercise load (1.00 BW, 1.25 BW, 1.50 BW), phase of movement (concentric, eccentric), position in the dynamic range of motion (flexion, mid, extension). Position in the dynamic range of motion was categorized as approximately 49–72 degrees for flexion, 25–48 degrees for mid, and 0–24 degrees for extension, which was approximately equivalent to 3.33-s intervals within each movement phase (concentric, eccentric). Lumbar extensor muscle surface EMG activity was evaluated for the effect of exercise load (1.00 BW, 1.25 BW, 1.50 BW), phase of movement (concentric, eccentric), position in range of motion (flexion, mid, extension) using analysis of variance (ANOVA) with repeated measures. Post hoc, pairwise comparisons were conducted using Tukey’s procedure, as needed. Statistical significance was set at alpha = 0.05. Stata 7.0 (Stata Corp., College Station, TX, USA) statistical package was utilized for all analyses.

## 3. Results

Participant characteristics and lumbar extension peak isometric torque are shown in Table 1. Peak torque values were generally within normal limits using normative data established by the manufacturer [21]. No serious adverse events were reported following isometric exercise testing on the lumbar dynamometer and dynamic exercise on the home back extension exercise device. 30% (3/10) of the participants reported delayed onset muscle soreness (DOMS) in the low back region that peaked 24–36 h after exercise, disappeared within 96 h, and did not affect physical function. All participants completed three repetitions of dynamic exercise at the three assigned loads and there was no indication that the loads were near maximal effort. 80% (8/10) of the participants displayed a progressive increase in lumbar extensor muscle surface EMG activity as exercise load was increased from 1.00 BW to 1.50 BW.

**Figure 3:**
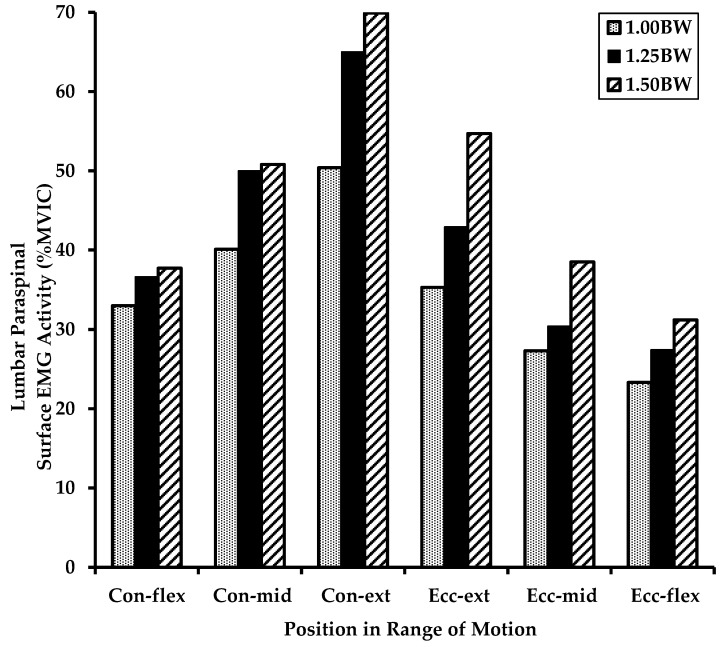
Graph of normalized lumbar extensor muscle surface EMG activity (in % MVIC) during dynamic exercise on a home back extension exercise device depicted by exercise load, phase of movement, and position in range of motion. Key: Mean values in % Maximum Voluntary Isometric Contraction (MVIC), BW = Bodyweight, Con = Concentric Phase, Ecc = Eccentric Phase, flex = flexion position in range of motion, mid = mid position, ext = extension position.

Normalized lumbar extensor muscle surface EMG activity by exercise load, phase of movement, and position in range of motion is shown in Table 2 and Figure 3. There was a significant effect of exercise load on lumbar extensor muscle surface EMG activity [F (2, 9) = 7.77, *p* = 0.0006]. Post-hoc analysis revealed that EMG activity at an exercise load of 1.50 BW was significantly greater than 1.00 BW (*p* < 0.05). There was a significant effect of phase of movement on lumbar extensor muscle surface EMG activity [F (1, 9) = 31.33, *p* < 0.0001], indicating that EMG activity was greater for the concentric phase compared to the eccentric phase. There was a significant effect of position in range of motion on lumbar extensor muscle surface EMG activity [F (2, 9) = 30.55, *p* < 0.0001]. Post hoc analysis revealed that EMG activity at the extension position was significantly greater than the mid position and flexion position (*p* < 0.05), and that EMG activity was greater at the mid position than the flexion position (*p* < 0.05).

## 4. Discussion

The findings of this study indicate that full range of motion lumbar extension exercise on a home back extension exercise device effectively activates the lumbar extensor muscles in a progressive manner. Supported by the findings of no adverse events, the study suggests that a home exercise device is well-tolerated and safe for lumbar extensor exercise training in adults without contraindications to resistance exercise. While DOMS was experienced by some participants, it is a typical response following unfamiliar lumbar extension exercise—whether isometric exercise tests or dynamic exercise training [3]. Nevertheless, educating clients and patients on expectations regarding likelihood of muscle soreness and stiffness is important.

The observed range of mean values for normalized lumbar extensor surface EMG activation levels suggests that the home back extension exercise device can provide gradual progressive resistance for the lumbar extensors. Furthermore, the mean value of the lowest observed activation level is likely low enough for patients during early phases of therapeutic exercise programs. Since the exercise loads for this study were arbitrarily selected, the observed lumbar extensor EMG activation levels do not necessarily represent the maximum attainable levels during exercise on the device. Given the progressive nature of the observed activation levels, it is possible that higher activation levels can be attained with additional external loads. Whether these activation levels provide the overload stimulus necessary for lumbar extension strength gains is unknown.

One explanation for the lack of progressive resistance for the lumbar extensors despite higher exercise loads in 20% of participants is that activation of the lumbar extensor muscles during compound trunk extension is variable [22,23]. Other trunk extensor muscles, such as the glutes and hamstrings, may be recruited at varying levels during compound trunk extension at different loads, which is consistent with previous research on other exercise devices [22,23]. The specific biomechanical strategies for individuals to generate force on the home back extension exercise device are unknown.

As expected, lumbar extensor muscle activation levels during exercise on the home back extension exercise device were higher during the concentric phase than the eccentric phase. A possible explanation for the wide variation of activation levels among the positions in the range of motion (i.e., nearly 100% greater in the extended position) is that isotonic exercise was performed (versus variable resistance exercise). While it is unknown if similar variations in lumbar extensor muscle activation exist during exercise on the computerized lumbar dynamometer and other trunk extension movements, this finding is consistent with the flexion-relaxation phenomena of the posterior lumbar musculature [24]. Therefore, slow, controlled movements emphasizing both concentric and eccentric phases throughout the full pain-free range of motion (particularly extension) while gradually increasing resistance over time is recommended for safety and optimal activation of the lumbar extensor muscles [3]. This recommendation is generally consistent with the guidelines of the American College of Sports Medicine for resistance exercise training of major muscle groups [25].

Many of the design characteristics to isolate the lumbar spine of the prototype home back extension exercise device that was tested in this study are similar to those of the computerized lumbar dynamometer, such as a footboard, femur restraint, and pelvic restraint pad (Figure 1 and Figure 2). However, the prototype device does not incorporate a lap belt. Without restraint from a lap belt, participants were able to hike (extend) their hips during the terminal extension phase of exercise, which may have permitted the hamstrings and gluteal muscles to elicit force production at the expense of the lumbar extensors [4,7].

Based on the findings of this study and other prototype testing, a new version of the device (Figure 2B) was developed and is currently available (Figure 2B). The current version has similar overall design features and addresses shortcomings of the prototype. For example, the current version uses hydraulic mechanisms to apply external loads and has a lap belt to enhance pelvic stabilization. These changes could help accommodate a wider range of exercise loads (both lower and higher) and improve isolation of the lumbar spine. There is no reason to expect that the improvements in the new version would negatively impact the ability to apply progressive resistance compared to the prototype tested.

### 4.1. Limitations

This study has some limitations that impact its generalizability. For example, the sample size was small and consisted of individuals without a history of clinical low back pain. The exercise loads were arbitrarily selected and are not representative of the full range of loads possible with the device. Future research is warranted to assess loading conditions at different ranges, such as those lower than body weight, in healthy individuals and those with low back pain. Also, the study did not assess longer-term exercise training programs. Moreover, future research would be useful in healthy individuals and patients with low back pain to compare the effectiveness of the home back extension exercise device with other exercise devices, such as the Variable Angle Roman Chair [26], on the ability to activate the lumbar extensor muscles, optimize strength gains, and enhance clinical outcomes.

### 4.2. Pragmatic Applications

The home back extension exercise device was able to safely administer progressive loads for the lumbar extensor muscles. Thus, trainers and clinicians can incorporate the device for delivering lumbar extensor exercise training programs in community-dwelling adults without contraindications to resistance exercise. The safety of lumbar extensor strengthening exercises has been documented and can be enhanced by starting the program at a low load and gradually applying progressive resistance at subsequent sessions [3]. Implementation of this device for home use outside of clinical settings does not preclude adequate supervision, which is needed to monitor safety, encourage proper movement, and improve adherence. Recent research suggested that education is needed to enhance acceptance of telehealth physical therapy by patients with chronic low back pain [27], and ongoing supervision provides an opportunity to do so. While numerous approaches for supervision are possible, supervision of a home exercise program using this device could be accomplished through an initial on-site orientation in the home setting followed by periodic virtual sessions hosted by a qualified professional. The Official Disability Guidelines (ODG) Medical Treatment Guidelines generally recommend lumbar extension exercise equipment (e.g., MedX lumbar extension machine) for the management of chronic low back pain [28]. The ODG indicates this modality may be an option for first-line treatment when implemented within a supervised physical therapy program, such as face-to-face in the clinic or virtually via telehealth [26]. Implementation of the home back extension exercise device appears to be appropriate for this purpose. Lumbar strengthening exercises and other exercises are recommended for the management of chronic low back pain [3,29]. However, subclassification of specific individuals for the management of low back pain to receive lumbar strengthening exercises or other exercises (e.g., motor control) has not been validated through research and is beyond the scope of this study. Thus, the role of lumbar strengthening exercises within an exercise program depends on client/patient preferences, functional goals, and trainer/clinician experiences [2,3]. If the goal is to strengthen the lumbar extensor muscles, then exercises that apply progressive loads to the lumbar extensor muscles should be implemented, such as the home back extension exercises assessed in this study.

## 5. Conclusions

The findings of this study indicate that dynamic exercise on a home back extension exercise device is safe and provides a mechanism to progressively activate the lumbar extensor muscles. This device can be used for progressive resistance exercise training programs for community-dwelling adults without contraindications to resistance exercise.

## Figures and Tables

**Table 1:** Participant demographic characteristics and lumbar extension torque values.

Variable	Total (*n* = 10)		Female (*n* = 5)		Male (*n* = 5)	
	Mean	SD	Mean	SD	Mean	SD
Age (year)	33.0	8.4	29.0	9.5	37.0	5.5
Body Height (cm)	174.0	6.9	170.7	8.1	177.3	3.3
Body Weight (kg)	76.2	18.4	62.3	9.8	90.2	13.4
Peak IM torque (N-m)	392.0	190.5	254.2	95.3	529.9	157.8

Key: Peak IM torque (N-m) = Peak lumbar extension isometric torque in Newton-meters (N-m) assessed on a dynamometer.

**Table 2:** Normalized lumbar extensor muscle surface EMG activity (in % MVIC) during dynamic exercise on a home back extension exercise device depicted by exercise load, phase of movement, and position in range of motion.

	Exercise Load					
	1.00 BW		1.25 BW		1.50 BW	
	Mean	SD	Mean	SD	Mean	SD
Full Repetition	34.9	16.0	42.1	11.8	47.1	9.8
Concentric Phase:						
Full Concentric Phase	41.2	17.9	50.5	16.2	52.8	10.3
Flexion Position	33.0	16.1	36.6	16.6	37.7	11.3
Mid Position	40.1	19.9	50.0	16.3	50.8	8.7
Extension Position	50.4	21.9	65.0	20.7	69.9	16.0
Eccentric Phase:						
Full Eccentric Phase	28.7	16.0	33.6	12.7	41.4	13.8
Flexion Position	23.3	14.9	27.4	12.7	31.2	13.0
Mid Position	27.3	16.8	30.4	13.4	38.5	12.3
Extension Position	35.3	18.5	42.9	14.4	54.7	27.8

Key: Values are in % Maximum Voluntary Isometric Contraction (MVIC), BW = Body Weight, SD = Standard Deviation.

## Data Availability

The data presented in this study are available on request from the corresponding author.
